# Novel Emerging Molecular Targets in Non-Small Cell Lung Cancer

**DOI:** 10.3390/ijms22052625

**Published:** 2021-03-05

**Authors:** Sara Elena Rebuzzi, Lodovica Zullo, Giovanni Rossi, Massimiliano Grassi, Veronica Murianni, Marco Tagliamento, Arsela Prelaj, Simona Coco, Luca Longo, Maria Giovanna Dal Bello, Angela Alama, Chiara Dellepiane, Elisa Bennicelli, Umberto Malapelle, Carlo Genova

**Affiliations:** 1 Medical Oncology Unit 1, IRCCS Ospedale Policlinico San Martino, 16132 Genoa, Italy; massigrassi.mg@gmail.com (M.G.); murianni.veronica@gmail.com (V.M.); 2 Department of Internal Medicine and Medical Specialties (Di.M.I.), University of Genoa, 16132 Genoa, Italy; tagliamento.marco@gmail.com (M.T.); carlo.genova@hsanmartino.it (C.G.); 3 Lung Cancer Unit, IRCCS Ospedale Policlinico San Martino, 16132 Genoa, Italy; lodozullo@gmail.com (L.Z.); simona.coco@hsanmartino.it (S.C.); luca.longo@hsanmartino.it (L.L.); mariagiovanna.dalbello@hsanmartino.it (M.G.D.B.); angela.alama@hsanmartino.it (A.A.); chiara.dellepiane@hsanmartino.it (C.D.); elisa.bennicelli@hsanmartino.it (E.B.); 4 Medical Oncology Department, Ospedale Padre Antero Micone, 16153 Genoa, Italy; giovanni.rossi.1689@gmail.com; 5 Department of Medical, Surgical and Experimental Sciences, University of Sassari, Via Roma 151, 07100 Sassari, Italy; 6 Department of Medical Oncology, Fondazione IRCCS Istituto Nazionale dei Tumori, 20133 Milan, Italy; arsela.prelaj@istitutotumori.mi.it; 7 Department of Electronics, Information, and Bioengineering, Polytechnic University of Milan, Piazza Leonardo da Vinci 32, 20133 Milan, Italy; 8 Department of Public Health, University of Naples Federico II, 80138 Naples, Italy; umbertomalapelle@gmail.com; 9 UO Clinica di Oncologia Medica, IRCCS Ospedale Policlinico San Martino, 16132 Genoa, Italy

**Keywords:** non-small cell lung cancer, targeted therapy, MET, RET, NTRK, KRAS, PIK3CA, HER2

## Abstract

In the scenario of systemic treatment for advanced non-small cell lung cancer (NSCLC) patients, one of the most relevant breakthroughs is represented by targeted therapies. Throughout the last years, inhibitors of the epidermal growth factor receptor (EGFR), anaplastic lymphoma kinase (ALK), c-Ros oncogene 1 (ROS1), and V-raf murine sarcoma viral oncogene homolog B (BRAF) have been approved and are currently used in clinical practice. However, other promising molecular drivers are rapidly emerging as therapeutic targets. This review aims to cover the molecular alterations with a potential clinical impact in NSCLC, including amplifications or mutations of the mesenchymal–epithelial transition factor (MET), fusions of rearranged during transfection (RET), rearrangements of the neurotrophic tyrosine kinase (NTRK) genes, mutations of the Kirsten rat sarcoma viral oncogene (KRAS) and phosphatidylinositol-4,5-bisphosphate 3-kinase, catalytic subunit alpha (PIK3CA), as well as amplifications or mutations of human epidermal growth factor receptor 2 (HER2). Additionally, we summarized the current status of targeted agents under investigation for such alterations. This revision of the current literature on emerging molecular targets is needed as the evolving knowledge on novel actionable oncogenic drivers and targeted agents is expected to increase the proportion of patients who will benefit from tailored therapeutic approaches.

## 1. Introduction

Since the discovery of actionable molecular alterations, the therapeutic approach of advanced non-small cell lung cancer (NSCLC) patients has been constantly evolving throughout the years. As the current paramount of molecularly driven treatment of NSCLC is represented by mutations of the epidermal growth factor receptor (*EGFR*) [1], rearrangements of the anaplastic lymphoma kinase (*ALK*) [2] or c-Ros oncogene 1 (*ROS1*) [3], as well as mutations of the v-Raf murine sarcoma viral oncogene homolog B (*BRAF*) [4], the identification of these genetic alterations is considered pivotal in current clinical practice worldwide. Nonetheless, other oncogenic drivers with therapeutic relevance have emerged more recently, and clinically active inhibitors have been approved or are under development.

This review aims to summarize the current state of the art and future directions of novel emerging therapeutic targets for NSCLC, with mention to the involved molecular pathways and the potential therapeutic strategies assessed in clinical studies. We performed a comprehensive literature search to collect relevant data on PubMed for published articles and the most relevant international conferences involving lung cancer from the American Association for Cancer Research (AACR), American Society of Clinical Oncology (ASCO), European Society for Medical Oncology (ESMO), and World Conference on Lung Cancer (WCLC), looking for the most updated and complete information.

## 2. MET

### 2.1. Epidemiology

Mesenchymal–epithelial transition factor (*MET*)-activating alterations, including overexpression (15–70%), amplification (2–5%), and exon 14 (*METex14*) skipping mutations (3–4%), are oncogenic drivers in 5–9% of newly diagnosed non-squamous NSCLC [5,6]. *MET* amplification is also responsible for the 10–20% of the acquired resistance, especially to EGFR inhibitors, but also to ALK inhibitors, although *METex14* mutations are also emerging as a resistance mechanism [7,8]. Alterations of *METex14* are generally mutually exclusive with other primary oncogenic drivers, except for *MET* amplifications and copy number variants [9].

Several studies showed that *MET*-positive patients are more frequently Caucasian, elderly (median age >70 years), female, never-smokers, and at an earlier stage compared with other oncogene-addicted NSCLC patients [10,11,12]. Alterations of *MET* are generally associated with aggressive disease, resistance to anticancer therapies, and a poor prognosis when not treated with MET inhibitors [11,13,14].

These aberrations have been identified mostly in the adenocarcinoma histology, especially with sarcomatoid and adenosquamous features (10–20%) [10,11]. In a retrospective analysis on radiological features of *METex*14-mutated NSCLC patients, the primary tumor seemed to present as solid and peripheral masses with a high frequency of multifocal and extrathoracic metastases, mainly to the bone, brain, and adrenal glands [15].

### 2.2. Molecular Pathway

The c-*MET* gene is a proto-oncogene located at chromosome 7q21-q31, which encodes for a heterodimer receptor tyrosine kinase (RTK), also known as hepatocyte growth factor receptor (HGFR), with extracellular, transmembrane, juxtamembrane, and kinase domains [9].

The binding of MET to its ligand HGF leads to its homodimerization with subsequent auto-phosphorylation and activation of the intracellular tyrosine kinase domains. The activation of c-MET stimulates many downstream signaling pathways, including mitogen-activated protein kinase (MAPK)/extracellular signal-regulated kinase (ERK), phosphatidylinositol 3-kinase (PI3K)/protein kinase B (AKT), mammalian target of rapamycin (mTOR), and Janus kinase (JAK)/signal transducer and activator of transcription (STAT) pathways [9,16]. These pathways are involved in cell survival, proliferation, migration, invasion, angiogenesis, and the epithelial-to-mesenchymal transition.

The *MET* exon 14 encodes the juxtamembrane domain containing the tyrosine 1003 (Y1003), which is the binding site of a ubiquitin protein (Cbl) that regulates ubiquitin-mediated c-MET receptor degradation. The *METex1*4 skipping mutations alter the splicing process, producing a MET variant that lacks Y1003, which consequently cannot bind Cbl. This alteration results in decreased degradation and subsequent ligand-independent MET activation [17].

### 2.3. Diagnostic Methodology

Many diagnostic techniques can be used for the detection of *MET* alterations, both as gene aberrations and protein expression. Immunohistochemistry (IHC) has been used to detect MET overexpression and in past clinical trials to select and stratify patients. However, the level of MET protein overexpression and its activity has been observed to be poorly correlated [18]. For this reason, MET IHC assessment has been replaced with other techniques. Gene amplifications are mostly detected by fluorescence in situ hybridization (FISH), but also DNA/RNA sequencing techniques are employed. However, there is no consensus on the definition of *MET* amplification since different studies have proposed various scores and different cut-offs [16]. *METex*14 mutation can be detected by DNA sequencing or RNA-based sequencing techniques, including next-generation sequencing (NGS), which is the primary detection method, NanoString, and quantitative real-time polymerase chain reaction (qRT-PCR) [16,18].

### 2.4. Therapeutic Implications

The two most clinically relevant *MET* alterations are *METex*14 skipping mutations as the primary oncogenic driver and *MET* amplification as acquired resistance to tyrosine kinase inhibitors (TKI). In these settings, many anti-MET drugs with different mechanisms of action, either as single agents or combinations, have been developed and are currently under investigation. They are classified into MET TKIs and monoclonal antibodies (anti-MET antibodies and anti-MET antibody-drug conjugates) [11].

Since data on MET-inhibitors are rapidly expanding, here we mainly discuss the major therapeutic strategies, referring for details to dedicated reviews and clinical trials’ results [5,9,10,11].

#### 2.4.1. MET as Primary Oncogenic Driver

MET TKIs are the principal MET-targeted drugs for *METex14* mutations and they are divided into non-selective (multi-targeted) TKIs and the more efficient selective TKIs. Among the first ones, the most studied TKI with positive results was crizotinib, whereas the second ones (e.g., capmatinib, tepotinib, savolitinib) have recently provided encouraging data [6] (Table 1). Due to the great number of MET inhibitors currently under investigation, in this review we focused on the Phase II–III trials of selective TKIs.

##### Crizotinib

In 2018, crizotinib received breakthrough therapy designation from the US Food and Drug Administration (FDA) based on its efficacy observed in the expansion cohort of the PROFILE 1001 trial on *METex14*-mutated advanced NSCLC patients [19,20]. More recently, the updated data reported an overall response rate (ORR) of 32% and a median progression-free survival (mPFS) of 7.3 months [21]. Many Phase I–II (e.g., AcSè, METROS) and retrospective analyses confirmed the reported activity and survival results [16,22,23,24]. In these studies, crizotinib reported similar results in *MET*-amplified patients. Therefore, crizotinib has been recommended for both *METex*14-mutated and *MET*-amplified NSCLC patients according to international guidelines [25].

##### Capmatinib

Capmatinib is the first FDA-approved selective MET-inhibitor in *METex*14-mutated NSCLC patients and its use is currently suggested in the first-line setting [25,26]. The approval (May 2020) was based on the efficacy reported by the multi-cohorts Phase II GEOMETRY mono-1 trial [27]. In this study, capmatinib was assessed as first- and subsequent-line in *MET*-dysregulated advanced NSCLC patients, showing a promising antitumor activity among *METex14*-mutated patients, especially untreated ones and those with brain metastases. In *MET*-amplified patients, the efficacy was higher in tumors with a high gene copy number. The randomized Phase III GeoMETry-III trial (NCT04427072) is currently ongoing to evaluate capmatinib compared to docetaxel in pre-treated NSCLC patients. The primary endpoint is mPFS and secondary endpoints included median overall survival (mOS), ORR, and median duration of response (mDOR). Ongoing trials are assessing capmatinib in *METex14*-mutated patients progressing to MET-inhibitors (NCT02750215) or systemic therapy for advanced disease (NCT04427072).

##### Tepotinib

The ongoing Phase II VISION study is evaluating the activity of tepotinib in NSCLC patients harboring *METex*14 mutations detected by liquid and tissue biopsies. Preliminary data were presented at the 2019 ASCO annual meeting and the final results have been recently published [28,29]: tepotinib has shown promising activity and long responses across treatment lines, including patients with brain metastases.

Based on the preliminary data, in September 2019 tepotinib obtained the FDA breakthrough therapy designation for metastatic *METex*14-mutated NSCLC patients that progressed after platinum-based chemotherapy [30].

##### Savolitinib

Savolitinib, a novel potent and selective MET-inhibitor that is under evaluation in a Phase II study on *METex14*-mutated NSCLC (NCT02897479), has demonstrated promising anti-tumor activity and a durable response in a preliminary analysis [31].

##### Other MET-Inhibitors

Other MET-TKIs are currently under evaluation in Phase II trials, including cabozantinib (NCT03911193, NCT01639508), merestinib (NCT02920996), bozitinib (NCT03175224, NCT04258033), and Glumetinib (NCT04270591) [9,10].

#### 2.4.2. MET as Secondary Acquired Resistance

Amplification of *MET* is the main resistance mechanism to EGFR-TKIs in NSCLC patients, both for the first- and second-generations (50–60%) and for the third-generation (15–20%) [17]. Several ongoing trials are investigating the role of MET-TKIs as a single agent or in combination with EGFR-TKIs, to overcome the acquired resistance to anti-EGFR therapies (Table 2). Therapeutic strategies included the addition of a MET-inhibitor to the previous EGFR-TKI. In the case of first- and second-generation EGFR-TKI, the exon 20 p.T790M mutation had not been present at disease progression. Capmatinib, cabozantinib, and tepotinib in combination with EGFR-TKIs have already yielded promising antitumor activity in *EGFR*-mutated, *MET*-amplified NSCLC patients [16,18,32].

#### 2.4.3. MET Alterations and Immunotherapy

The *MET* activation has shown to induce programmed cell-death protein-1–ligand-1 (PD-L1) expression, contributing to immune suppression and evasion [33]. Several retrospective analyses have reported controversial results on the predictive value of *MET* alterations to immunotherapy [10,17]. In the IMMUNOTARGET study, 36 *MET*-positive NSCLC patients receiving immunotherapy experienced limited activity (ORR 16%) and survival outcomes (mPFS and mOS of 3.4 and 18.4 months, respectively), similar to other analyses [34,35]. However, durable responses were also observed by other studies in the same setting [36,37].

Immunotherapy generally seemed to have less anti-tumor activity in *MET*-positive NSCLC patients compared with TKIs and chemotherapy. Moreover, PD-L1 and tumor mutational burden (TMB) seemed not to predict response to immunotherapy in these patients [9]. Therefore, immunotherapy should be considered only after failure of other effective treatments, although the role of immune-based combinations in this setting needs further investigation [17]. In this context, several Phase I–II trials on the combination of MET-TKIs and immunotherapy are ongoing (e.g., NCT02323126, NCT03468985).

## 3. RET

### 3.1. Epidemiology

Alterations of the rearranged during transfection (*RET*) gene in NSCLC are detected in approximately 2% of patients [38,39,40]. Overall, *RET* aberrations are more represented in adenocarcinoma histology. Patients with lung cancer and *RET* involvement are frequently never-smokers and young. These neoplasms can present a solid pattern with signet rings or might otherwise be characterized by a lepidic pattern. The primary tumor is often small but with early lymph node involvement. Alterations of *RET* and other NSCLC driver aberrations were initially thought to be mutually exclusive, but there are consistent data on the presence of concomitant gene alterations in a small percentage of cases [40,41].

### 3.2. Molecular Pathway

The *RET* gene is a proto-oncogene located on chromosome 10q11.2 that encodes for a transmembrane receptor tyrosine kinase (RTK), consisting of an extracellular domain, a hydrophobic transmembrane domain, and an intracellular domain, including a juxtamembrane portion, a tyrosine kinase domain, and carboxy-terminal tail. Activation of *RET* is given from the interaction of the glial cell line-derived neurotrophic factor (GDNF) family receptor α (GFRα) co-receptor with ligands belonging to the family of neurotrophic factors, including GDNF [42], neurturin (NRTN) [43], persephin (PSPN) [44], and artemin (ARTN) [45]. The physiological ligands of RET are part of the growth factors of the glia; indeed, RET is physiologically present in the neuroectodermal tissues and is crucial for the development of the fetus, in particular for the genesis of the enteric parasympathetic nervous system [46]. The interaction between the receptor and its ligands ultimately leads to the formation of homodimers and the phosphorylation of tyrosine residues of the kinase domain. The phosphorylation of the SH domain activates downstream signaling pathways, such as RAS/MAPK/ERK, PI3K/AKT, and JAK/STATs, which, in turn, lead to signal transduction for cell proliferation, differentiation, and migration.

To date, several fusion partner genes of *RET* have been discovered, the most frequent being the rearrangement with kinesin family member 5B gene (*KIF5B-RET*) of which there are seven variants, coiled-coil domain containing 6 (*CCDC6–RET*), nuclear receptor coactivator 4 (*NCOA4-RET*), and tripartite motif-containing 33 (*TRIM33–RET*) [47]. Irrespective of the gene partner and the breakpoint, *RET* fusions lead to the formation of a new protein that includes the coiled-coil domain of the partner gene and RET intracellular kinase domain, which always retains the tyrosine kinase activity. The coil-coiled domain of the partner gene leads to a ligand-independent RET dimerization, resulting in its constitutive activation.

### 3.3. Diagnostic Methodology

The diagnostic methods to detect *RET* fusions are still under debate. More specifically, IHC has demonstrated a low specificity in detecting *RET* rearrangements [39,48,49] and it is not considered applicable in clinical practice. Conversely, high sensitivity and specificity are guaranteed by NGS, FISH, and qRT-PCR techniques. The NGS approach, both whole genome and whole transcriptome sequencing, represents the best technology for discovering novel *RET* rearrangements, although it is still significantly expensive; moreover, it necessitates computational expertise and infrastructures, preventing its application in a diagnostic context. Currently, the standard for diagnosing *RET* fusions is represented by FISH [49]. This technique is highly sensitive and able to detect fusions independently of the partner genes, although it cannot define which fusion gene is involved. Nonetheless, it requires experienced personnel for data interpretation, and, to date, there is presently no agreement on the cutoff to be used to define the positivity of *RET*. Conversely, RT-PCR has the great advantage over FISH of identifying the fusion partner gene. Besides, it can provide a diagnosis even on cytological samples, compared to FISH that needs histological samples [50]. Unlike FISH, a possible limitation is that RT-PCR cannot trace unknown or novel fusion partners, resulting in an underestimation of the prevalence of *RET* rearrangements [51]. Consequently, these two techniques have often been combined as screening methods.

### 3.4. Therapeutic Implications

Previous studies have shown that *RET* fusions are associated with a lower expression of thymidylate synthase [41], which is linked to high sensitivity to treatment with pemetrexed, with an ORR reaching up to 40%. In the last year, two targeted therapies have shown an important activity in these patients, surpassing the results achieved by other TKIs, so far. Indeed, selpercatinib and pralsetinib (Blue 667) have received FDA approval for the treatment of patients with *RET* fusion in NSCLC, due to their activity and efficacy. Previously, vandetanib, lenvatinib, sorafenib, cabozantinib, and alectinib, reached in small studies an ORR ranging from 16 to 50% and an mPFS that did not exceed 6 months [52,53,54,55]. LIBRETTO-001, a Phase III trial of selpercatinib for the treatment of NSCLC patients, showed an ORR of 64% in pretreated patients, with an mPFS of 17.5 months. The trial enrolled also previously untreated patients. In the subgroup of 39 patients who received selpercatinib as first-line treatment, the ORR was 85% [56]. Pralsetinib is another drug approved in 2020 by the FDA for the treatment of NSCLC patients based on the results of the phase I/II ARROW trial. Pralsetinib demonstrated an ORR of 57% in pretreated patients while it reached 70% in previously untreated patients, a response that lasted at least 6 months in 80% of pretreated patients and 58% of naïve patients [57] (Table 3).

## 4. NTRK

### 4.1. Epidemiology

Alterations of the neurotrophic tyrosine kinase (*NTRK*) genes (i.e., *NTRK1*, *NTRK2*, and *NTRK3*) are rare in NSCLC, representing less than 1% (about 0.1–0.6%) of the NSCLC population [5,58,59]. Although the proportion is very small, the global incidence of NSCLC patients is high, making these relatively few cases a relevant number of patients in absolute terms. Given the rarity and the relatively recent discovery of this target, any solid data on morphological and clinical characteristics of NSCLC with *NTRK* involvement are still lacking.

The largest study on NSCLC retrospectively included 4872 cases and identified only 11 cases with *NTRK1-3* fusions. Of these cases, nine were adenocarcinoma, including two mucinous and one with neuroendocrine features, while one was a squamous cell carcinoma (SCC), and one a large-cell neuroendocrine carcinoma [59].

High expression of the protein encoded by *NTRK2* (TrkB) and of its ligand, namely brain-derived neurotrophic factor (BDNF), are the only proteins found to be correlated with a higher prevalence of vascular invasion, lymph nodes metastases, and advanced stage in NSCLC patients. These factors resulted in a poorer prognosis for this population [60,61].

The other relevant clinical characteristic that has emerged is that the majority of *NTRK*-driven NSCLC patients were never-smoker (about 80%), as it is usually observed for other kinase fusion-positive NSCLCs [62].

Interestingly, different studies highlighted that *NTRK* fusions seem to be almost mutually exclusive with alterations in other known genes, such as *ALK*, *ROS-1*, *MET*, and *RET* [63,64].

### 4.2. Molecular Pathway

The *NTRK* genes encode the TrkA, TrkB, and TrkC transmembrane glycoproteins, respectively, that together with their natural ligands, which are the nerve growth factor (NGF), BDNF, neurotrophin-3 (NT-3), and NT-4, are involved in the physiological development and function of the central and peripheral nervous systems [59,60,61]. Fusions of *NTRK* genes lead to overexpression of Trk proteins and, therefore, to the constitutive activation of downstream signaling pathways such as RAS/MAPK, PI3K/AKT, and PLC-γ, responsible for cancer cells transformation, proliferation, and survival [5,61]. The most commonly detected fusions are ETS Variant Transcription Factor 6 (*ETV6*)-*NTRK3* and Echinoderm Microtubule Associated Protein Like 4 (*EML4*)-*NTRK3*, although more than 50 other fusion partners are currently known [65].

Interestingly, another role for one of these transmembrane proteins has been recently found. Indeed, preclinical studies have shown that the increased expression of TrkB promotes the suppression of E-cadherin expression and enhances the activity of the matrix metalloproteinase-2 (MMP-2) in lung SCC cells, promoting cancer aggressiveness [60,61].

### 4.3. Diagnostic Methodology

The *NTRK* fusions can be studied with different methods, among which IHC is one of the most widespread used and characterized by high sensitivity (95 to 100%) and specificity (93% to 100%); although these data derive from very small studies, this technique can be considered a good screening tool as of today, given the rarity of *NTRK* gene alterations [58,62].

Another relevant diagnostic tool is represented by FISH, which may be helpful when the histologic tumor type is known to frequently harbor an *NTRK* fusion, although this may not be the best option in NSCLC due to its aforementioned low prevalence [59,62]. Nowadays, the employment of newly approved targeted therapies may be either approved for patients with an *NTRK* gene fusion diagnosed by NGS techniques or within clinical trials. IHC testing can be accepted but needs to be validated with another diagnostic method, thus resulting in a more time-consuming procedure for each patient. Thus, the NGS approach may be proposed either as front-line or after positivity at the IHC screening as it is recommended in the latest ESMO guidelines [66].

### 4.4. Therapeutic Implications

The discovery of actionable *NTRK* gene fusions represented a revolution in oncology. The first-generation TRK inhibitors entrectinib and larotrectinib received, in fact, an agnostic approval by the FDA for patients with *NTRK* fusion-positive solid tumors because of their impressive results in Phase I/II trials [5,59].

Entrectinib is a TRKA, TRKB, TRKC, ROS1, and ALK multi-inhibitor, and data from three Phase I/II trials (ALKA-372-001, STARTRK-1, and STARTRK-2) reported an ORR for NSCLC patients of 70–75% with 10% of complete responses (CR), an mDOR of 12.9 months, and an mOS of 23.9 months (Table 4) [67,68,69]. Larotrectinib, by contrast, is a pan-TRK inhibitor and a recently published pooled analysis of three Phase I/II trials showed, among NSCLC patients, an ORR of 75%, an mPFS of 28.3 months, and an mOS of 44.4 months [70].

These two drugs are both very effective and very well tolerated with few side effects. The main difference is the central nervous system (CNS) penetration as entrectinib can cross more effectively the blood–brain barrier. As a matter of fact, entrectinib has proved its efficacy in patients with brain metastases, although it has also shown some CNS side-effects, such as dizziness [67,68,69].

Across time, as in many other targeted therapies, the tumor can develop acquired resistance, but new generations of TRK inhibitors, such as taletrectinib (TPX-0005), selirectinib (LOXO-195), and repotrectinib, are currently under evaluation in ongoing Phase I/II clinical trials [5,59].

## 5. KRAS

### 5.1. Epidemiology

Kirsten Rat Sarcoma viral oncogene (*KRAS*) mutations are among the most frequently detected oncogenic drivers in NSCLC. These mutations were identified in lung cancer almost three decades ago and have been traditionally associated with a poor prognosis compared to *KRAS* wild type tumors [71]. *KRAS* mutations were found in 26% of NSCLC, almost exclusively in adenocarcinomas and in the smoker population [72]. Only a minority of never smokers (6%) harbors the *KRAS*-mutant NSCLC. Smoking history significantly increases the chance to detect *KRAS* mutation in lung cancer, regardless of pack-years of smoking [73].

### 5.2. Molecular Pathway

*RAS* genes, including *KRAS*, are proto-oncogenes encoding intracellular guanine nucleotide-binding proteins that belong to the GTPase family. RAS proteins are composed of a catalytic domain called “G domain”, which binds guanine nucleotides and activates signaling, and a hypervariable region (HVR), which determinates where the RAS proteins are localized on the cell membrane to perform their signaling function. The downstream signaling depends on the RAS bound state: the guanosine triphosphate (GTP)-bound state is the active form while the guanosine diphosphate (GDP)-bound state is the inactive form [74]. GTP/GDP cycling is regulated by guanine nucleotide exchange factors (GEFs) and GTPase-activating proteins (GAPs); GEFs promote RAS activation by binding RAS and causing GDP separation. Otherwise, GAPs stimulate the intrinsic GTP hydrolysis activity of RAS to increase inactive RAS–GDP forming [75]. The RAS–GTP complex activates multiple signaling cascades in response to extracellular signals. Some of these pathways include RAF/MEK/ERK, PI3K/AKT/mTOR, RalA/B, and TIAM1/RAC1, which regulate cell proliferation, differentiation, and apoptosis. Most *RAS* mutations usually involve exons 2 and 3, impairing the conversion from GTP-bound to GDP-bound state and constitutively activating the downstream signaling. The most frequent mutations are the nucleotide substitutions in 12 codons of *KRAS* exon 2 p.G12C, p. G12D, and p.G12V [74].

### 5.3. Diagnostic Methodology

Currently, the evaluation of *KRAS* gene alterations is strongly recommended in patients with colon–rectal cancer or NSCLC eligible for anti-EGFR therapy [25]. Many studies have indeed demonstrated that *KRAS* mutations are negative biomarkers of response to anti-EGFR. This mechanism of resistance derives from the persistent stimulation of the EGFR/RAS/RAF/ERK/MEK pathway when *KRAS* is mutated, regardless of the mutational status of *EGFR*. The *KRAS* testing is usually performed on tumor tissue, rather than from secondary lesions in case of metastatic disease. There are more than 60 methods currently available for *KRAS* testing, including sequencing, high-resolution melting analysis (HRM), single-strand conformation polymorphism (SSCP), denaturing gradient gel electrophoresis (DGGE), denaturing high-performance liquid chromatography (DHPLC), array/strip analysis, and allele-specific PCR [76].

### 5.4. Therapeutic Implications

Over the past few years, several efforts have been done to find a predictive role of *KRAS* alterations. While data from chemotherapy response are conflicting, some trials have shown a correlation between *KRAS* mutations and the benefit from immune checkpoint inhibitors [77]. A meta-analysis by Kim et al. demonstrated that immune checkpoint inhibitors therapy significantly improved OS in patients with *KRAS*-mutant NSCLC but not in those with the *KRAS* wild-type tumor [78]. The biological rationale is probably related to an increased expression of PD-L1 and high tumor immunogenicity [79]. However, co-occurring serine/threonine kinase (*STK11*) and *KRAS* mutations seem to be associated with PD-1 inhibitor resistance [80].

To date, the standard of care for *KRAS*-driven tumors is the same as that for non-oncogene addicted NSCLC since no targeted therapies are approved. Different strategies to directly target KRAS or its downstream effectors have been developed, either as a single agent or in combination with other TKIs, chemotherapy, and immunotherapy (Table 5).

Several preclinical and clinical trials have investigated the inhibition of multiple KRAS pathways but most of them failed in giving significant survival or response improvements [74].

Recent developments of novel targeted therapies against *KRAS* exon 2 p.G12C have shown encouraging results. These molecules irreversibly bind to *KRAS* exon 2 p.G12C in the GDP-bound state, preventing the conversion to the active form and impairing the downstream signaling [79]. Preliminary data have demonstrated anti-tumor activity with durable responses and a favorable safety profile [81,82]. Currently, a multicenter Phase III clinical trial of sotorasib (CODEBREAK200) in previously treated advanced *KRAS*-mutant NSCLC is ongoing (NCT04303780).

An additional therapeutic strategy has been explored with the use of cyclin-dependent kinase (CDK) 4/6 inhibitors, as a synthetic lethal interaction between *KRAS* oncogenes and *CDK4* in NSCLC [83]. An ongoing Phase III clinical trial (JUNIPER) is evaluating the efficacy and safety of abemaciclib compared to erlotinib in previously treated patients with advanced *KRAS*-mutated NSCLC. The study did not meet its primary endpoint of OS but demonstrated an improvement in PFS and ORR [84].

## 6. PIK3CA

### 6.1. Epidemiology

The phosphatidylinositol-4,5-bisphosphate 3-kinase, catalytic subunit alpha (*PIK3CA*) gene encodes for the catalytic subunit alpha of the phosphoinositide 3-kinase (PI3K) protein. Mutations of *PIK3CA* in lung cancer are uncommon, being prevalently detected in SCC histology, while its amplification is conversely frequently found [85]. The most common mutations are reported in exon 9 and 20. In tumor samples from 1144 NSCLC patients, *PIK3CA* mutations had an incidence of 3.7%, raised to 8.9% if counting only SCC [86], while in another cohort of 1117 NSCLCs, they were detected in 3.9% of SCCs and 2.7% of adenocarcinomas [87]. Nevertheless, PI3K/AKT/mTOR signaling is one of the main activated intracellular downstream pathways in different cancer types. The prognostic value of *PIK3CA* mutations is still partially undefined since survival analyses from different studies were discordant [86,87]. Nevertheless, a recent meta-analysis correlated the presence of *PIK3CA* mutations with worse OS and PFS in patients with NSCLC [88]. This uncertainty is probably related to the co-occurrence of alterations other than those involving PI3K; a genomic sequencing of specimens from early-stage NSCLC revealed *PIK3CA* as the most frequently mutated gene in co-existence with *EGFR* and *KRAS* mutations [89]. Patients harboring *PIK3CA* mutations are more commonly current or former smokers, although they were also observed in never smokers [87]. Notably, *PIK3CA* mutations are more frequently detected in metastases than in the primary tumor [90]. Thus, PIK3CA-mutated NSCLC represents a clinically and genetically heterogeneous subgroup [91].

### 6.2. Molecular Pathway

The PI3Ks are a family of lipid kinases able to phosphorylate the 3’-OH group of phosphatidylinositols and phosphoinositides on the cell membrane. These kinases are heterodimers with a catalytic (p110) and regulatory (p85) subunit, grouped in classes; class IA proteins, mainly related to human cancer, are usually activated by growth factor receptors, such as EGFR, insulin growth factor 1-receptor (IGF1-R), and HER2/neu. Such receptors represent the upstream signal of PI3K, leading to the activation of the catalytic domain of p110, which phosphorylates the phosphatidylinositol bisphosphate (PIP2) to phosphatidylinositol triphosphate (PIP3); subsequently, PIP3 locates AKT to the plasma membrane by binding to the phosphorylated lipid products [92]. Phosphatase and tensin homologue deleted on chromosome 10 (PTEN) acts as an inverse regulator through PIP2 and PIP2 dephosphorylation. AKT is a serine/threonine protein able to phosphorylate and consequently activate/inactivate numerous downstream pathways involving cytoplasmic and nuclear substrates that regulate apoptosis, cell cycle, cell survival, and proliferation, among which nuclear factor kappa-light-chain-enhancer of activated B cells (NFκB) transcription factor is one of the most important [92]. mTOR is a serine/threonine kinase complexed with other proteins to form the mTORCH complexes. mTORC2 promotes the activation of AKT via phosphorylation at serine 473 [91], while mTORC1 phosphorylates the p70S6 kinase (S6K1) and the eukaryotic initiation factor 4E binding protein 1 (4EBP1), leading to increased protein synthesis and cell growth [93].

### 6.3. Diagnostic Methodology

Currently, the evaluation of *PIK3CA* gene alterations is not recommended in clinical practice since no targeting drugs have been approved to date for lung cancer treatment. Activating mutations in *PIK3CA* can be detected in tissue or plasma specimens. Diagnostic testing within clinical trials is usually performed using NGS (see studies in Table 6).

### 6.4. Therapeutic Implications

PI3K inhibitors can be classified into pan-PI3K inhibitors [94,95,96,97,98,99,100,101,102], PI3K and mTOR inhibitors [103], and selective PI3K inhibitors [104]. The molecular downstream pathways can also be blocked by single inhibition of AKT and mTOR.

Table 6 reports the main characteristics of clinical studies investigating the role of PI3K inhibitors in the treatment of advanced stage lung cancer. Several other PI3K-targeting agents, not reported in this review, have been tested at pre-clinical or clinical level, with inconsistent results.

## 7. HER2

### 7.1. Epidemiology

Three main alterations involving the human epidermal growth factor receptor 2 (HER2) can be identified: *HER2* gene mutations, *HER2* gene amplification, and HER2 protein overexpression.

#### 7.1.1. HER2 Mutations

Mutations of *HER2*, mostly represented by exon 20 insertion, account for a minor number of cases of adenocarcinomas of the lung (2 to 4%) [105] and are generally associated with a particular clinical phenotype presentation. Indeed, similarly to *EGFR* exon 20 insertions, they are usually found in younger patients, never smokers, and in patients with a smaller tumor size. Conversely, no relationship with sex, lymph nodes involvement, or tumor stage has been reported, so far. Additionally, unlike *EGFR* mutations, the prevalence of *HER2* insertions appears to be comparable between Caucasian and Asian populations. Mutations in *HER2* appear to be mutually exclusive with other drivers of genetic alterations. Furthermore, *HER2* represents an independent poor prognostic factor and, similarly to *EGFR* insertions, is intrinsically resistant to the currently available therapies [106,107].

#### 7.1.2. HER2 Amplification and Protein Expression

Up to now, the biologic relationship of HER2 mutations, amplifications, and protein overexpression has not definitively been elucidated. HER2 amplification and HER2 overexpression (defined as a high level (3+) by IHC) have been detected in 2% to 5% and 2% to 4% of lung cancer, respectively. This confusion forcefully discloses during patient selection phase of clinical trials testing HER2-targeted therapies for NSCLC [108].

On this matter, in 2015 Li et al. assessed the presence of HER2 alterations on tumor samples from 175 patients with recurrent/stage IV lung adenocarcinomas. HER2 amplification and HER2 mutations were detected in 5/175 and 4/148 patients (27 cases had no evaluable specimen), respectively, including three patients with an identical 12-base-pair insertion (p.A775_G776insYVMA; c.2324_2325ins12) in exon 20 and one with an unspecified 9-base-pair insertion in exon 20. Forty-six patients showed a polysomy (HER2 copy number ≥ 4 but HER2-to-CEP17 ratio <2). No match was found between four mutations and amplifications and no HER2 overexpression was identified [109]. Other studies confirmed no overlapping between HER2 mutations and HER2 amplification in NSCLC, enhancing the assumption that they represent two different cancerogenic entities [105,109].

### 7.2. Molecular Pathway

HER2 belongs to an RTK family that includes EGFR (ERBB1), HER2 (ERBB2/NEU), HER3 (ERBB3), and HER4 (ERBB4). HER2 is a 185 kDa transmembrane glycoprotein encoded by the *ERBB2* gene located at chromosome 17q. As mentioned above, dysregulation of HER2 in NSCLC can be caused by mutations, amplification, and protein overexpression [110,111]. The amplification of *HER2* promotes tumorigenesis and is involved in the pathogenesis of several human cancers. In contrast to EGFR, no ligand has yet been recognized for HER2. Still, HER2 seems to be the main involved partner in the dimerization of all ERBB family components [112]. After the ligand binds to the extracellular domain of the receptor, HER2 undergoes heterodimerization, inducing a HER2 tyrosine kinase activation through phosphorylation of the intracellular tyrosine kinase domain, leading to activate several downstream signaling pathways. These include the RAS–RAF–MEK–ERK (MAPK) and the PI3K–AKT–mTOR pathways, which are involved in cell survival, proliferation, and apoptosis [113].

### 7.3. Diagnostic Methodology

Currently, there are different methods to diagnose HER2 overexpression, including IHC, Western blot, and enzyme-linked immunosorbent assay (ELISA), whereas, FISH, silver in situ hybridization (SISH), chromogenic in situ hybridization (CISH), PCR, Southern blot, and NGS [114] are employed to detect HER2 amplification [115]. Finally, HER2 mutations are detected by NGS, Sanger sequencing, qRT-PCR, and matrix-assisted laser desorption ionization–time of flight mass spectrometry (MALDI-TOF MS) [116]. Approximately 80% of HER2 evaluations start with IHC as a screening test. Among these tests, IHC and FISH constitute the current standard to assess HER2 expression and *HER2* amplification, respectively. While IHC is a quick and cost-effective test [117], FISH is a highly sensitive and specific assay [118]. Finally, sequencing methods are probably the most used approaches for detecting *HER2* mutation [119]. However, except for qRT-PCR, all the other approaches can also detect the exact amino acid positions.

### 7.4. Therapeutic Implications

Among new generation anti-HER2 for *HER2* mutation the most promising therapeutic strategies are reported below and in Table 7.

In the ASCO 2020 Meeting, results from the DESTINY-Lung01 multicenter Phase II trial were presented. Forty-two patients diagnosed with relapsed/refractory NSCLC *HER2* mutation were treated with an anti-HER2 antibody conjugate called fam-trastuzumab deruxtecan-nxki (T-DXd; Enhertu). Promising results reported an ORR of 61.9% (1 CR, 25 PR and 12 SD) and an mPFS of 14.0 months (95% CI, 6.4–14.0), with a not reached mDOR (95% CI, 5.3–not evaluable) and OS. The most common serious (Grade ≥ 3) drug-related adverse events (AEs) included neutropenia (26.2%) and anemia (16.7%), with particular attention to an AE of special interest such as interstitial lung disease in 11.9% with G2 severity [120]. Based on these results, a breakthrough therapy designation has been granted from the FDA for the T-DXd drug in *HER2*-mutant NSCLC and gastric cancer [121].

Poziotinib is an oral, irreversible pan-HER TKI, initially investigated in the Asian population. In the ESMO 2020 Congress, ZENTITH20-2, a multicenter phase II trial, reported results from a cohort of 90 *HER2* mutated patients. ORR was 35.1% (95% CI: 24.4–47.1%) and 27.8% (95% CI: 18.9–38.2%) in 74 evaluable patients and in all 90 patients, respectively. Median PFS and mDORe were 5.5 months (range: 0.03–13.1+ months) and 5.1 months (range: 1–12.3+ months), respectively. Patients with brain metastases obtained an ORR of 28.6% [122]. The most common treatment-related grade ≥3 AEs were rash (30%), diarrhea (26%), and mucosal inflammation (14%). Results from the first expanded access program of 30 *EGFR*/*HER2* insertion reported a higher ORR for *HER2* insertion compared to *EGFR* insertion patients (50% vs. 19%), with no differences in mPFS (5.6 months, CI95% 3.6–7.1 months) [123].

Pyrotinib is another oral, irreversible pan-HER TKI against HER1, HER2, and HER4 for HER2+ (immunochemistry expression) tested in NSCLC [124]. In 2019, Gao et al. published the first results of an open-label, multicenter, single-arm Phase II study. Sixty pretreated NSCLC patients with *HER2* insertion were enrolled, whereas patients with active brain metastases or those already treated with an anti-HER2 targeted therapy were excluded. The ORR was 31.7% (95% CI; 20.3–45.0), while the PFS was 6.8 months (95% CI 4.1–8.3). Diarrhea was reported as the only treatment-related grade 3 AE, occurring in ≥2 patients [125].

Among the new-generation TKIs, mobocertinib (TAK788) is a small-molecule oral TKI designed to selectively target both *EGFR* insertion and *HER2* mutation. Exciting results from an ongoing Phase I/II NCT02716116 trial including both *EGFR* insertion and *HER2* mutation in different cohorts pushed the FDA for a breakthrough therapy designation for *EGFR* insertion. Results from ongoing studies on *HER*-mutated patient cohorts are awaited [126].

Lastly, in 2019, results from the multicenter single-arm Phase II NICHE trial were published by Dziadziuszko et al., testing afatinib, an EGFR inhibitor, in 13 NSCLC patients pre-treated with platinum-based CHT harboring *HER2* mutations. Afatinib did not show the expected disease control, with a median PFS of 15.9 weeks (95% CI: 6.0–35.4) and OS of 56 weeks (95% CI; 16.3 weeks –NR). Moreover, severe drug-related AEs were reported: dyspnea (15.3%), acute renal injury (7.6%), and mucositis (7.6%) [127].

Several other drugs, such as trastuzumab and emtansine, have also demonstrated a role in *HER2*-altered NSCLC, but none of them has demonstrated a practical clinical application so far.

Therefore, the definition of “HER2+ NSCLC” is insufficient, as it does not exhaustively distinguish the complexity of this gene alterations. Indeed, the failure of many clinical trials aimed at targeting HER2 may have been derived from considering these alterations as a single entity [127,128,129]. Conversely, the DESTINY-Lung01 trial, which distinguished between HER2 overexpression and *HER2* mutation in the two different cohorts, is a good example of a successful trial. In Table 7 we reported almost all historical trials that investigated different agents against *HER2* alterations.

## 8. Discussion

Throughout the last decade, the treatment scenario of advanced NSCLC patients has been undergoing a revolution in terms of therapeutic targets. Notably, EGFR represents a paradigm of targetable oncogenic drivers, with a constant increase in terms of translational knowledge and clinical applications; moreover, the subsequent concept of acquired resistance mechanisms, which in turn might become therapeutic targets, is leading the management of *EGFR*-mutated NSCLC beyond its initial limits [1]. Even though the history of other actionable oncogenic drivers has been shorter and slower compared to *EGFR*, a similar progress is being achieved, such as the identification of acquired resistance mutations during first- or second-generation ALK inhibitors, and the publication of data suggesting variable sensitivity to the available inhibitors based on the specific reported mutation [130].

**Figure 1:**
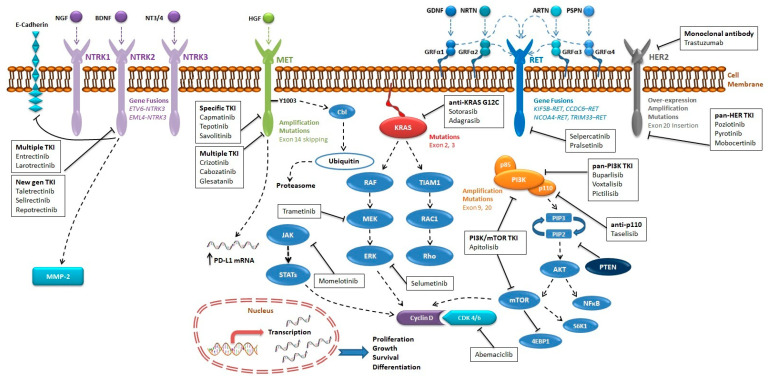
Molecular pathways of novel emerging targets in NSCLC and agents in clinical development. Several molecular pathways are physiologically activated by the interactions between circulating growth factors (colored circles) and trans-membrane receptors (colored sticks crossing the cell membrane), which result in the downstream activation of intracellular proteins (colored ovoids) associated with cell proliferation, increased aggressiveness, or immune escape. The details for each individual pathway are described in the appropriate paragraphs. Molecular alterations (e.g., gene fusions) potentially associated with tumorigenesis are reported next to the relevant molecule. When available, investigational agents able to inhibit specific pathways are reported (blank boxes), with reference to the specifically targeted molecule or interaction.

In this context, the identification of additional oncogenic drivers that can be exploited in the management of advanced NSCLC patients is expected to significantly increase the proportion of patients receiving targeted therapy (Figure 1).

This is particularly relevant, as the current percentage of patients with clinically actionable drivers in the Caucasian population accounts for 15–20% of non-squamous NSCLC (considering *EGFR*, *ALK*, *ROS1*, and *BRAF*), and the potential novel emerging targets might further increase this proportion. While the approval of any *KRAS* exon 2 p.G12C-specific inhibitor would potentially result in a further 10–15% of the patients with non-squamous NSCLC becoming eligible for targeted therapy (the same proportion currently eligible for EGFR inhibitors), the individual impact of each other driver seems more limited. Nonetheless, even if the relative number of patients harboring a specific molecular alteration may represent a little percentage of the whole NSCLC population, testing multiple actionable oncogenic drivers is expected to have a huge impact, because of the relevant absolute numbers of NSCLC patients. Moreover, since patients harboring oncogenic drivers might have an underwhelming clinical benefit from immune checkpoint inhibitors, their identification is decisive to choose the best therapeutic strategy for each patient [34].

An additional subject of discussion is represented by the clinical outcomes observed with targeted therapies. Notably, for several agents, as aforementioned, only Phase I–II data have been generated so far; however, at least for some of these drivers (e.g., *MET*, *RET*, *NTRK*), the observed outcomes have been consistently encouraging, with deep and prolonged objective responses, including intracranial responses. Moreover, the magnitude of clinical benefit appears to be more pronounced if eligible patients receive targeted therapies early, possibly as a first-line treatment.

As a matter of fact, the increased knowledge on actionable genomic targets and the development of effective drugs translates into the necessity to guarantee the widest possible accessibility, both in terms of molecular testing and access to novel compounds.

With regards to molecular testing, the most appropriate assays should be employed, taking into account both the increasing number of evaluated targets and the limited amount of tumor sample available through small biopsies or cell blocks, as well as the time needed to process such samples. The first steps regarding molecular analyses for clinical purposes have been represented for years by single-gene testing: to date, due to the increasing number of tests, such an approach is becoming impractical for NSCLC management. In this setting, the progressive shift towards diagnostic platforms able to analyze multiple genes at once, such as NGS technology, appears to be the next mandatory step for molecular biology laboratories involved in oncology, provided that the necessary resources and skills are optimized, and that the most appropriate sequencing tests are offered to each patient.

Once the tests have been performed and any actionable mutation identified, one additional issue might be represented by accessibility to appropriate targeted agents. Indeed, the rate of approval of novel agents might be different among different countries, especially when different regulatory agencies are involved, such as the FDA and the European Medicines Agency (EMA); as a result, living in different countries may lead to unequal accessibility and disparities. Possible ways to address this issue are represented by clinical trials, which are usually multi-institutional, international studies, as well as expanded access programs, which are designed to provide early access to innovative drugs; based on such information, both approaches should be highly encouraged in cancer centers.

Such is the relevance of properly organized molecular biology tests in oncology and subsequent access to targeted therapies, that several comprehensive cancer centers are focusing on the development of multi-disciplinary Molecular Tumor Boards, designed to provide governance while addressing any disparity in terms of access to oncogenic-driven assays and drugs [131].

## 9. Conclusions

While acknowledged oncogenic drivers, such as *EGFR*, *ALK*, *ROS1*, and *BRAF*, have a solid role in the therapeutic algorithms for advanced NSCLC, other actionable targets are progressively being identified, and novel targeted antineoplastic agents are being developed. The most promising novel targets are represented by *MET*, *RET*, and *NTRK*, although other drivers, namely *HER2* and *PIK3CA*, might experience relevant updates in the near future. With regards to *KRAS*, while this driver has been widely known for years, only recent clinical trials have generated encouraging data, and might eventually result in targeted therapy for a relatively high proportion of NSCLC patients.

Finally, while additional oncogenic targets are being identified, an effort to guarantee accessibility to appropriate molecular testing and novel drugs is a priority in the current setting of NSCLC management.

## Figures and Tables

**Table 1:** Principal single-arm Phase I–II trials on MET-TKI in METex14-mutated advanced NSCLC patients.

Drug	Trial	Phase	Therapy Line *	*N* pts	Main Results	Status
*Multikinase inhibitors*						
Crizotinib	PROFILE-1001 (NCT00585195)	I	1L ≥2L	Tot 69 26 in 1L 43 in ≥2L	ORR tot = 32% ORR 1L = 25% ORR 2L = 37% mDOR = 9.1 mo mPFS = 7.3 mo mOS = 20.5 mo	Closed FDA-Approved
	AcSé trial (NCT02034981)	II	≥1L	25	ORR = 40% mDOR = 2.4 mo mPFS = 3.6 mo mOS = 9.5 mo	Closed
	METROS (NCT02499614)	II	≥2L	9	ORR = 20% mDOR = NE mPFS = 2.6 mo mOS = 3.8 mo	Closed
*MET-specific TKI*						
Capmatinib	GEOMETRY-mono-1 (NCT02414139)	II	1L 2L,3L	28 in 1L	ORR = 68% mDOR = 12.6 mo mPFS = 12.4 mo	Ongoing FDA-Approved
				69 in 2L,3L	ORR = 41% mDOR = 9.7 mo mPFS = 5.4 mo	
Tepotinib	VISION (NCT02864992)	II	1L,2L,3L	66 diagnosed with LBx	ORR = 48% mDOR = 9.9 mo mPFS = 8.5 mo	Ongoing (preliminary data) FDA breakthrough designation
				60 diagnosed with TBx	ORR = 50% mDOR = 15.7 mo mPFS = 11.0 mo	
				99 diagnosed with LBx + TBx	ORR = 46% mDOR = 11.1 mo mPFS = 8.5 mo	
Savolitinib	NCT02897479	II	≥2L	70	ORR = 47.5% mDOR = NR mPFS = 6.8 mo	Ongoing

MET—mesenchymal-epithelial transition factor; TKI—tyrosine kinase inhibitor; 1L—first-line; 2L—second-line; 3L—third-line; N—number; pts—patients; LBx—liquid biopsy; TBx—tissue biopsy; *ORR*—overall response rate; mo—month; mDOR—median duration of response; NE—not estimable; mPFS—median progression-free survival; mOS—median overall survival; NR—not reached; FDA—Food and Drug Administration. An asterisk (*) indicates the line or lines of treatment for advanced NSCLC in which the investigational agent or regimen was employed in each reported trial.

**Table 2:** Principal Phase Ib–II trials on MET-TKI in MET-amplified EGFR-mutated advanced NSCLC patients.

Drug	Trial	Phase	Therapy Line *	Prior Treatment	Treatment Arm	*N* pts	Main Results	Status
*Single agent*								
Capmatinib	GEOMETRY -mono-1 (NCT02414139)	II	≥2L	NA	Capmatinib	69	ORR = 29% mDOR = 8.3 mo mPFS = 4.1 mo	Ongoing
*Combinations*								
Tepotinib	INSIGH (NCT01982955)	Ib/II	2L	1G-2G EGFR-TKIs	Tepotinib + Gefitinib vs. Platinum-based CT	19	ORR = 67% vs. 43% mPFS = 16.6 vs. 4.2 mo mOS = 37.3 vs. 13.1 mo	Closed
Capmatinib	NCT01610336	II	2L	Gefitinib/ Erlotinib	Capmatinib + Gefitinib	100	ORR = 47%	Closed
Cabozantinib	NCI 9303 II (NCT01866410)	II	≥2L	1G, 2G, 3G EGFR-TKIs	Cabozantinib + Erlotinib	37	ORR = 10.8% mPFS = 3.6 mo mOS = 13.1 mo	Closed

2L—second-line; NA—not available; 1G—first-generation; 2G—second-generation; EGFR—epidermal growth factor receptor; TKI—tyrosine kinase inhibitor; CT—chemotherapy; N—number; pts—patients; ORR—overall response rate; mo—month; mDOR—median duration of response; mPFS—median progression-free survival; vs.—versus; mOS—median overall survival. An asterisk (*) indicates the line or lines of treatment for advanced NSCLC in which the investigational agent or regimen was employed in each reported trial.

**Table 3:** Principal single-arm Phase I–II trials on TKI in RET-rearranged advanced NSCLC patients.

Drug	Trial	Phase	Therapy Line *	*N* pts	Main Results	Status
Selpercatinib	LIBRETTO-001 (NCT03157128)	II	≥1L	39 in 1L	ORR = 85% 1y-PFS = 75%	Closed FDA approved
				105 in ≥2L	ORR = 64% mDOR = 17.5 mo mPFS = 16.5 mo 1yPFS = 66%	
Pralsetinib	ARROW (NCT03037385)	I/II	≥1L	79	ORR = 56%	Ongoing FDA approved
Vandetanib	LURET (UMIN000010095 **)	II	≥2L	19	ORR = 53%	Ongoing (preliminary data)
Lenvatinib	NCT01877083	II	≥1L	25	ORR = 16%	Ongoing (preliminary data)
Sorafenib	000007515 **	II	≥2L	3	ORR = 0%	Closed
Cabozantinib	NCT01639508	II	≥1L	26	ORR = 28% mDOR = 7 mo mPFS = 5.5 mo mOS = 9.9 mo	Closed

1L—first-line; 2L—second-line; N—number; pts—patients; ORR—overall response rate; 1y-PFS—progression-free survival at one year; mo—month; mDOR—median duration of response; mPFS—median progression-free survival; mOS—median overall survival; FDA—Food and Drug Administration. An asterisk (*) indicates the line or lines of treatment for advanced NSCLC in which the investigational agent or regimen was employed in each reported trial. Two asterisks (**) indicate the UMIN trial number.

**Table 4:** Principal single-arm Phase I–II trials on NTRK inhibitors in NTRK-fused advanced NSCLC patients.

Drug	Trial	Phase	Therapy Line *	*N* pts	Main Results	Status ***
Entrectinib	ALKA-372-001 (EudraCT2012–000148–88)	I	Any	10 **	ORR = 70% mDOR = 12.9 mo mPFS = 14.9 mo mOS = 23.9 mo	Closed
	STARTRK-1 (NCT02097810)	I	Any			Closed
	STARTRK-2 (NCT02568267)	II	Any			Ongoing
Larotrectinib	LOXO-TRK-14001 (NCT02122913)	I	Any	12 **	ORR = 75% mDOR = NE mPFS = 28.3 mo mOS = 44.4 mo	Closed
	LOXO-TRK-15003 (NCT02637687)	I/II	Any			Ongoing
	NAVIGATE (NCT02576431)	II	Any			Ongoing

N—number; pts patients; ORR—overall response rate; mo—month; mDOR—median duration of response; mPFS—median progression-free survival; mOS—median overall survival; NE—not estimable. An asterisk (*) indicates the line or lines of treatment for advanced NSCLC in which the investigational agent or regimen was employed in each reported trial. Two asterisks (**) indicate that the studies were pooled analyses. Three asterisks (***) indicate that Entrectinib and Larotrectinib FDA approved for *NTRK*-fused solid tumors.

**Table 5:** Principal Phase Ib–III trials on TKI in KRAS-mutated advanced NSCLC patients.

Drug	Trial	Phase	Therapy Line *	Treatment Arm	*N* pts	Main Results	Status
*Targeting KRAS pathway*							
Selumetinib (MAPK inhibitor)	SELECT-1 (NCT01933932)	III	2L	Selumetinib + Docetaxel vs. Placebo + Docetaxel	510	mPFS = 3.9 vs. 2.8 mo mOS = 8.7 vs. 7.9 mo ORR = 20.1% vs. 13.7% mDOR = 2.9 vs. 4.5 mo	Closed
	IND.219 (NCT02337530)	II	1L	ARM A: Salumetinib intermittent + Pemetrexed/Platinum	20	ORR = 35% mPFS = 7.5 mo	Closed
				ARM B: Salumetinib *continuous* + Pemetrexed/Platinum	21	ORR = 62% mPFS = 6.7 mo	
				ARM C: Pemetrexed/Platinum	21	ORR = 24% mPFS = 4.0 mo	
Trametinib (MEK 1-2 inhibitor)	NCT01362296	II	≥2L	Trametinib vs. Docetaxel	134	mPFS = 12 vs. 11 weeks mOS = 8 mo vs. NR ORR = 12% vs. 12%	Closed
Momelotinib (JAK1-2 inhibitor)	NCT02258607	Ib	≥2L	Momelotinib + Trametinib	21	ORR = NR DCR = 57.1% mPFS = 3.6 mo mOS = 7.4 mo	Closed
Defactinib (FAK inhibitor)	NCT01951690	II	≥2L	Defactinib	55	12wks-PFS = 28%	Closed
*Targeting KRAS G12C*							
Sotorasib (AMG510)	CODEBREAK 100 (NCT03600883)	I/II	≥2L	Sotorasib	129 (59 NSCLC)	ORR = 32.2% DCR = 88.1% mPFS = 6.3 mo	Ongoing
Adagrasib (MRTX849)	KRYSTAL-1 (NCT03785249)	I/II	≥1L	Adagrasib	110 (79 NSCLC)	ORR = 45% DCR = 96%	Ongoing
*Targeting CDK 4/6*							
Abemaciclib (LY2835219)	JUNIPER (NCT02152631)	III	≥2L	Abemaciclib vs Erlotinib	453	mOS = 7.4 vs. 7.8 mo mPFS = 3.6 vs. 1.9 mo ORR = 8.9% vs. 2.7% DCR = 54.5% vs. 31.7%	Ongoing

1L—first-line; 2L—second-line; vs.—versus; N—number; pts—patients; mPFS—median progression-free survival; mOS—median overall survival; mo—month; ORR—overall response rate; DCR—disease control rate; mDOR—median duration of response; 12wks-PFS—progression-free survival at 12 weeks; NR—not reached. An asterisk (*) indicates the line or lines of treatment for advanced NSCLC in which the investigational agent or regimen was employed in each reported trial.

**Table 6:** Principal Phase I–II trials on PI3K or PI3K/mTOR inhibitors in PI3K-mutated advanced NSCLC patients.

Drug	Trial	Phase	Therapy Line	Treatment Arms ***	*N* pts	Main Results	Status
*Selective PI3K inhibitor* (*anti p110*α)							
GDC-0032 (Taselisib)	Lung-MAP sub-study (NCT02785913)	II	≥2L *	Taselisib	26 Sq	ORR = 4.8% mPFS = 2.9 mo mOS = 5.9 mo	Closed at futility analysis
*Pan-PI3K inhibitor*							
BKM120 (Buparlisib)	BASALT-1 (NCT01297491)	II	≥2L *	Buparlisib	30 Sq 33 Nsq	12 wks PFS = Sq 23.3%, Nsq 20.0% mOS = Sq 7.98 mo, Nsq 7.2 mo ORR = 3.3% Sq, 3.0% Nsq	Closed at futility analysis
	BASALT-2 (NCT01820325)	Ib/II	1L	Buparlisib + CBDCA + paclitaxel	6 Sq	ORR = 16.7%	Early study termination
	BASALT-3 (NCT01911325)	Ib/II	≥2L *	Buparlisib + docetaxel	27 Sq	ORR 80 mg/daily = 6% ORR 100 mg/daily = 18%	Early study termination
	NCT01487265	II	≥2L **	Buparlisib + erlotinib	37	3 mo PFS = 50.4% mOS = 12.2 ORR = 5.4%	Completed
XL765 (SAR245409, Voxtalisib)	NCT01390818	Ib	Any line	Voxtalisib + pimasertib (MEK inhibitor)	Advanced solid tumors (33 NSCLC)	ORR = 5%	Completed
	NCT00777699	I	≥2L **	Voxtalisib + erlotinib	Advanced solid tumors (37 NSCLC)	SD as best response	Completed
PX-866	NCT01204099	II	2L-3L	Docetaxel + PX-866 vs. docetaxel	95	ORR = 6% vs. 0%, *p* = 0.12 mPFS = 2.0 vs. 2.9 mo, *p* = 0.65 mOS = 7.9 vs. 9.4 mo, *p* = 0.9	Completed
XL147 (SAR245408)	NCT01392924	I	Further lines	XL147	Advanced solid tumors (24 NSCLC)	ORR = 16.7%	Completed
	NCT00692640	I	≥2L **	XL147 + erlotinib	Advanced solid tumors (20 NSCLC)	ORR = 3.7% DCR = 51.9%	Completed
GDC-0941 (Pictilisib)	NCT01458067	Ib	1L	Arm A: pictilisib + CBDCA + paclitaxel Arm B: CBDCA + paclitaxel + bevacizumab Arm C: CDDP + pemetrexed + bevacizumab Arm D: CDDP + pemetrexed	66	ORR = 43.9%	Completed
*Dual PI3K/mTOR inhibitor*							
GDC-0980 (Apitolisib)	NCT01301716	Ib	1L	Arm A: apitolisib + CBDCA + paclitaxel ARM B: CBDCA + paclitaxel + bevacizumab Arm C: CDDP + pemetrexed	Advanced solid tumors (39 NSCLC)	ORR = 64%	Completed

1L–first line; 2L–second line; 3L–third line; pts–patients; Sq–squamous; Nsq–non-squamous; CBDCA–carboplatin; CDDP–cisplatin; DCR–disease control rate; ORR–objective response rate; mPFS–median progression-free survival; mOS–median overall survival; NSCLC–non-small-cell lung cancer; RP2D–recommended phase 2 dose; EGFR TKI–epidermal growth factor tyrosine kinase inhibitor; MTD–maximum tolerated dose; SD–stable disease; NR–not reached. When patients with solid tumors were included, specific data for NSCLC were reported. * Disease progression to prior platinum-based chemotherapy; ** disease progression to EGFR TKI; *** indicates the line or lines of treatment for advanced NSCLC in which the investigational agent or regimen was employed in each reported trial.

**Table 7:** Principal single-arm Phase I–II trials on TKI in HER2-positive advanced NSCLC patients.

Drug	Trial	Phase	Therapy Line *	*N* pts	Main Results	Status
Trastuzumab deruxtecan (T-DXd)	DESTINY-Lung01 (NCT03505710)	II	≥2L	42 (HER2exp, HER2mut)	ORR = 61.9% mPFS = 14 mo	Ongoing
Poziotinib	ZENITH20-2 (NCT03318939)	II	≥2L	90 (HER2mut)	ORR = 27.8% mPFS = 5.5 mo	Ongoing
Afatinib	NICHE (NCT02369484)	II	≥2L	13 (HER2mut)	ORR = 7.7% mPFS = 15.9 wks mOS = 56 wks	Completed
Pyrotinib	NCT02834936	II	≥2L	60 (HER2mut)	ORR = 31.7% mPFS = 6.8 mo mOS = NA	Unknown
Mobocertinib (TAK-788)	NCT02716116	I/II	≥1L	57 (EGFR exon20ins = 39 HER2 mut = 13)	NA	Ongoing
Trastuzumab Emtansine	NCT02675829	II	≥1L	18 (HER2 mut)	ORR = 44% mPFS = 5 mo	Ongoing
Trastuzumab	HOT1303-B (UMIN000012551)	II	≥2L	10 (HER2exp, HER2mut)	ORR = 0% mPFS = 5.2 mo	Completed
Dacomitinib	NCT00818441	II	≥2L	26	ORR = 12% mPFS = 3 mo mOS = 9 mo	Completed

1L—first-line; 2L—second-line; N—number, pts—patients; HER2exp—HER2 expressing; HER2mut—HER2 mutated; ORR—overall response rate; wks—weeks; mo—month; mPFS—median progression-free survival; mOS—median overall survival; NA—not available. An asterisk (*) indicates the line or lines of treatment for advanced NSCLC in which the investigational agent or regimen was employed in each reported trial.

## Data Availability

No new data were created or analyzed in this study. Data sharing is not applicable to this article.
